# Diagnostic performance of kinetic parameters derived from ultrafast breast MRI in characterizing benign and malignant breast lesions: the added value of the semiautomatically based parameters

**DOI:** 10.1186/s13244-025-02162-8

**Published:** 2025-12-22

**Authors:** Rasha Karam, Farah A. Shokeir, Ali H. Elmokadem, Ahmed Abdallah, Omar Hamdy, Dalia Bayoumi

**Affiliations:** 1 Department of Radiology, Mansoura University, El Mansoura, Egypt; 2 Oncology Center, Mansoura University, El Mansoura, Egypt

**Keywords:** Magnetic resonance imaging, Breast cancer, Kinetics, Ultrafast

## Abstract

**Objectives:**

This study aimed to evaluate the efficacy of two combined ultrafast breast MRI kinetic parameters, combination 1 including time to enhancement [TTE], maximum slope [MS], and initial enhancement phase [IE phase] compared to combination 2 including relative enhancement [RE], maximum enhancement [ME], maximum relative enhancement [MRE], time to peak [TTP], and wash in rate in characterizing benign and malignant breast lesions.

**Materials and methods:**

This prospective study included 264 female patients with 273 breast lesions. The ultrafast protocol was done using the TWIST sequence. The parameters for combination 1 were generated manually; however, the parameters for combination 2 were generated semi-automatically. The overall performance of the ultrafast protocol was compared to the conventional dynamic MRI protocol.

**Results:**

The ultrafast protocol was obtained in 77 s. The mean interpretation time was 5 ± 2.7 and 1 ± 0.5 min for combinations 1 and 2, respectively. Combination 1 showed an AUC of 0.910, a sensitivity of 76.5% and a specificity of 90%, while combination 2 showed an AUC of 0.869, a sensitivity of 76.5%, and a specificity of 85% in differentiating benign from malignant lesions. Upon combining all parameters, the AUC, sensitivity, and specificity in discriminating between the two groups increased to 0.944, 80.4%, and 85%, respectively. Both ultrafast techniques and conventional MRI demonstrated excellent performance in discriminating between benign and malignant lesions (AUC = 0.921 vs 0.940, respectively).

**Conclusion:**

Adding the semiautomatically generated parameters derived from ultrafast breast MRI can improve the performance in characterizing breast lesions.

**Critical relevance statement:**

By studying ultrafast-derived semiautomatic, easily applicable parameters, we aim to reduce the acquisition and interpretation times of breast MRI without compromising performance, when used as a problem-solving modality in indeterminate breast lesions to characterize them as either benign or malignant.

**Key Points:**

- Adding semiautomatic ultrafast parameters to the MS and TTE improves the overall performance in characterizing breast lesions.
- The combined ultrafast parameters provide the highest discriminating power between benign and malignant breast lesions.
- Ultrafast MRI showed comparable performance to conventional dynamic contrast-enhanced MRI in the discrimination between benign and malignant breast lesions.

**Graphical Abstract:**

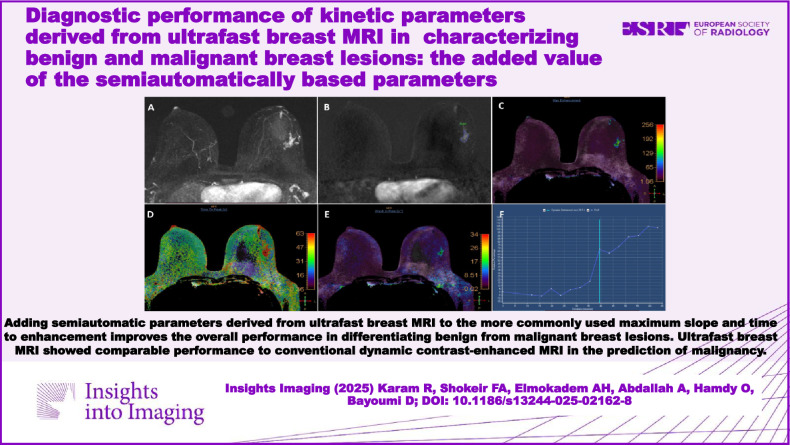

## Introduction

Breast MRI is known for its high sensitivity and negative predictive value (NPV) [1]. In limited situations, when other imaging modalities fail to characterize breast lesions, MRI can be used as a problem-solving tool [2]. Despite the benefits of breast MRI, it has many limitations, including long examination time, patient discomfort, and high cost [3–5]. To overcome these barriers, several abbreviated protocols have been recently introduced into practice. One of them is the ultrafast protocol, which was first introduced by Hermann et al They studied a sequence called time-resolved angiography with stochastic trajectories (TWIST), allowing for the construction of images at high temporal resolution [6, 7]. Later, other researchers reported that similar results can be obtained by using other techniques. However, they found that these complex sequences required longer reconstruction time [8]. Different ultrafast techniques share the same idea that early post-contrast images document the early inflow of contrast within the lesion without increasing acquisition time [6].

Mann et al dynamically used ultrafast sequences, allowing the production of an early wash-in curve, from which they first introduced a parameter called maximum slope (MS) as a tool to characterize breast lesions [8]. Since then, many studies have utilized other parameters, such as time to enhancement (TTE), and reported high specificity in predicting malignancy [5].

It was reported that the efficacy of adding early enhancement kinetics provided by the ultrafast protocol is at least equal to the efficacy of adding the washout parameter derived from the conventional dynamic curve [9, 10]. Thus, assessing early enhancing kinetics may help in better characterizing breast lesions without increasing acquisition time [11].

This study aims to evaluate the effect of adding ultrafast semiautomatically generated parameters (relative enhancement (RE), maximum enhancement (ME), maximum relative enhancement (MRE), time to peak (TTP), and wash-in rate), along with other commonly used parameters (TTE, MS, and initial enhancement (IE) phase), in differentiating between benign and malignant breast lesions. We also aimed to evaluate the overall diagnostic performance of ultrafast breast MRI compared to the conventional dynamic contrast-enhanced MRI (DCE-MRI) in characterizing breast lesions.

## Materials and methods

### Study population

This prospective, single-center, institutional review board-approved study (ethics committee reference number: R.23.05.2174) was conducted between August 2022 and September 2023 in female patients. The inclusion criteria include patients presenting with breast lesions, either detected clinically or by sonography or mammography. All patients’ radiological, clinical, and pathological data were reviewed after obtaining their written informed consent. Exclusion criteria include pediatric patients younger than 18 years old, patients with a previous history of breast cancer, and patients with a contraindication to MRI or gadolinium-based contrast media. All breast lesions included in this study underwent histopathological examination, which was obtained by ultrasound-guided trucut biopsy. The radiological results were correlated with histopathological results.

### Sample size calculation

Sample size calculation was based on the mean MS values in benign and malignant lesions retrieved from previous research [12]. Using the G Power program version 3.1.9.7 to calculate the sample size based on the effect size of 0.50, using a 2-tailed test, α error = 0.05, and power = 98%, the total calculated sample size will be 260 patients.

### MR protocol

An MRI was performed using a 1.5-Tesla MR system (Siemens Magnetom Aera) with a 16-channel breast coil. First, a non-contrast volume interpolated breath-hold examination (VIBE) series was obtained to create a subtraction series derived from the conventional DCE-MRI. Then, an ultrafast study was performed between the pre-contrast study and the first post-contrast sequence in the DCE-MRI protocol using the TWIST sequence in an axial plane without fat suppression.

The initial TWIST sequence consists of the initial pre-contrast phase followed by 14 post-contrast TWIST series after the injection of 0.1 mmol/kg of gadolinium contrast, which had a temporal resolution of 4.32 s each. Subtraction series of the ultrafast study were obtained by subtracting the 1st non-contrast TWIST frame from each post-contrast TWIST frame. 15 maximum intensity projection (MIP) images were reconstructed from these subtraction images.

The ultrafast study was followed by the DCE-MRI that consisted of five sequential post-contrast series done in the axial plane using the VIBE sequence.

The acquisition parameters used for both ultrafast TWIST sequence and DCE-MRI VIBE sequence were summarized in Table 1.

**Table 1:** Acquisition parameters for both Ultrafast MRI and conventional DCE-RI

	Ultrafast MRI	Conventional DCE-MRI
Sequence name	TWIST	VIBE
TR/TE	3.14/1.2	6/2 s
Flip angle	10 degree	20–25 degrees
FOV	300 × 300 mm^2^	300 × 300 mm^2^
Slice thickness	3.5 mm	2 mm
Acquisition time	77 s	10 min

*TWIST* time-resolved angiography with stochastic trajectories, *VIBE* volume interpolated breath-hold examination, *FOV* field of view

### Ultrafast MRI interpretation

**Fig. 1:**
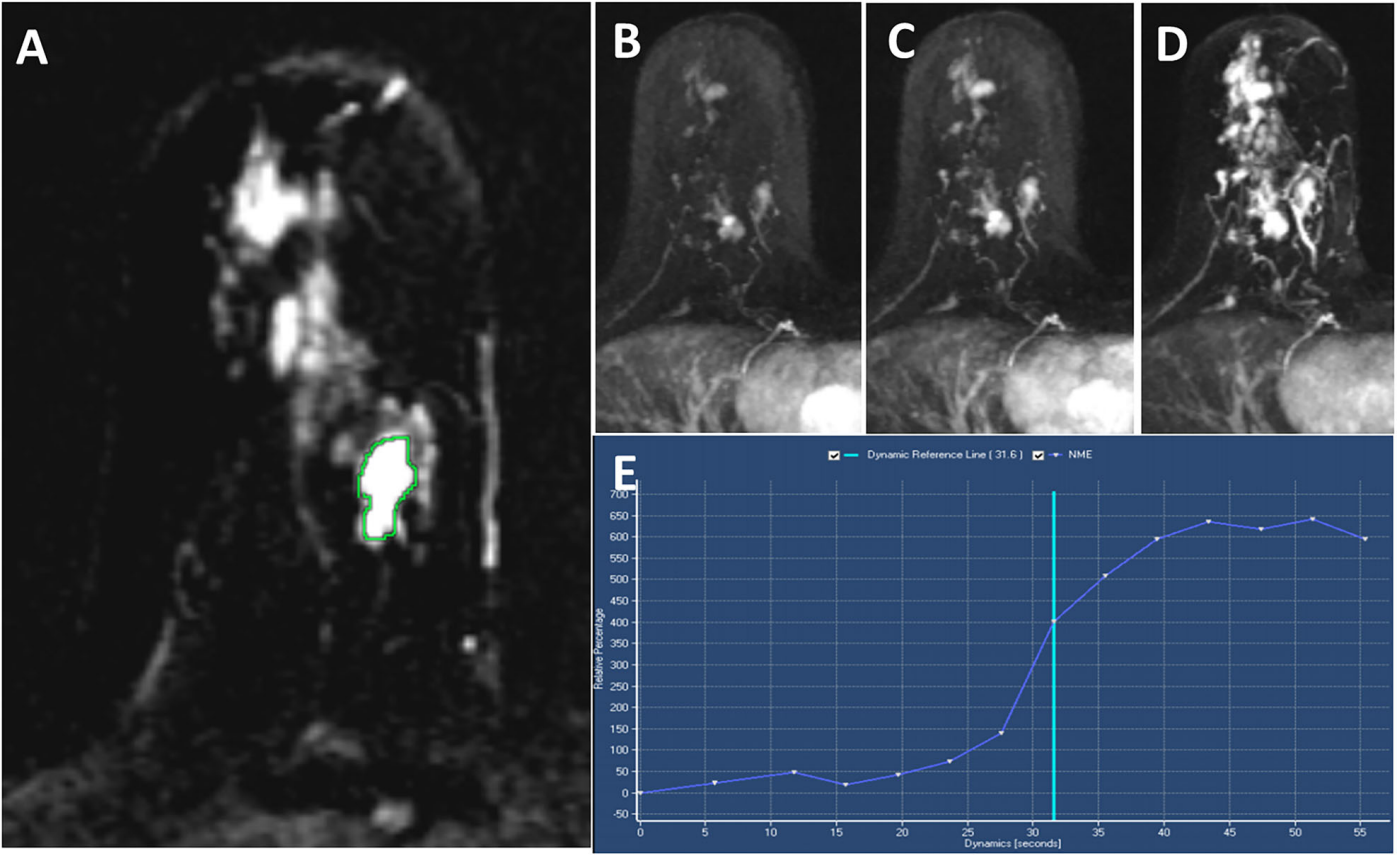
A 42-year-old female presented with a right breast lump (**A**), subtracted ultrafast image at phase 14 showing ROI placement within the mass. **B**–**D** Dynamic sequential MIP images derived from the ultrafast breast MRI (at phases 8, 10, and 12, respectively). **D** The phase of maximum lesion enhancement (phase 12) was used to assess the lesion’s morphology, showing multicentric, irregular-shaped masses in the lower quadrant of the right breast. **E** Wash-in curve derived from ultrafast sequences revealed TTE = 0 s; MS = 65%/s; IE phase = 0; RE = 635%; ME = 314; MRE = 640%, TTP = 51 s; wash in rate = 32/s. The lesions were IDC on histopathological analysis

In the first reading session, two radiologists with 10- and 14-year experience in breast imaging separately interpreted the ultrafast images while they were blinded to the final pathological results. The phase at which the lesions showed maximum visual enhancement on the subtraction and MIP images was used to assess the lesion’s morphologic data. Meanwhile, the last phase of MIP images was used to assess the background parenchymal enhancement (BPE), as it was the phase of maximum parenchymal enhancement. A free hand region of interest (ROI) was manually put lining the outlines of the whole lesion on the subtracted images at the phase in which the lesion showed ME visually as shown in (Fig. 1); the non-enhancing areas (areas of cystic degeneration, necrosis, and hemorrhage) were excluded, then a wash-in ultrafast curve was automatically generated by the workstation (extended MR 130 Workspace 2.6.3.5, Philips Medical Systems).

**Fig. 2:**
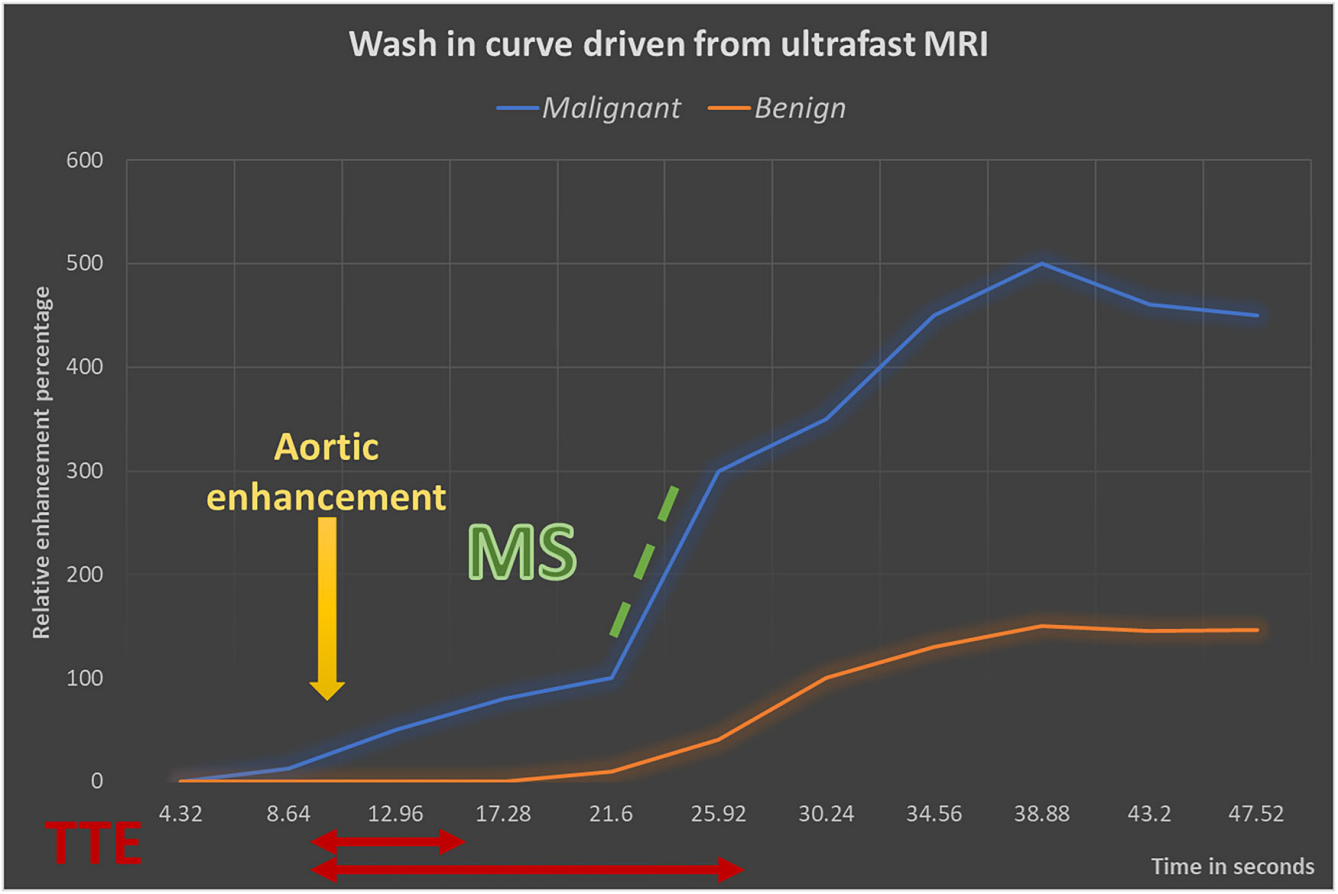
An example of the wash-in curve of benign (orange curve) and malignant (blue curve) lesions derived from ultrafast sequences. MS was calculated manually by first choosing the steepest part of the curve, then calculating the difference between the RE percentages (dashed green lines) divided by the time interval (dashed in purple lines). TTE was calculated visually from the subtracted MIP images as the time interval between the beginning of the descending aortic enhancement and lesion enhancement (red lines, short line for malignant lesion and long line for benign lesion). In this example, the aorta began to enhance at 4.32 s, and the malignant lesion started to enhance at 8.64 s; however, the benign lesion began to enhance at 17.28 s, resulting in a TTE for the malignant lesion of 4.32 s and for the benign lesion of 12.96 s

**Fig. 3:**
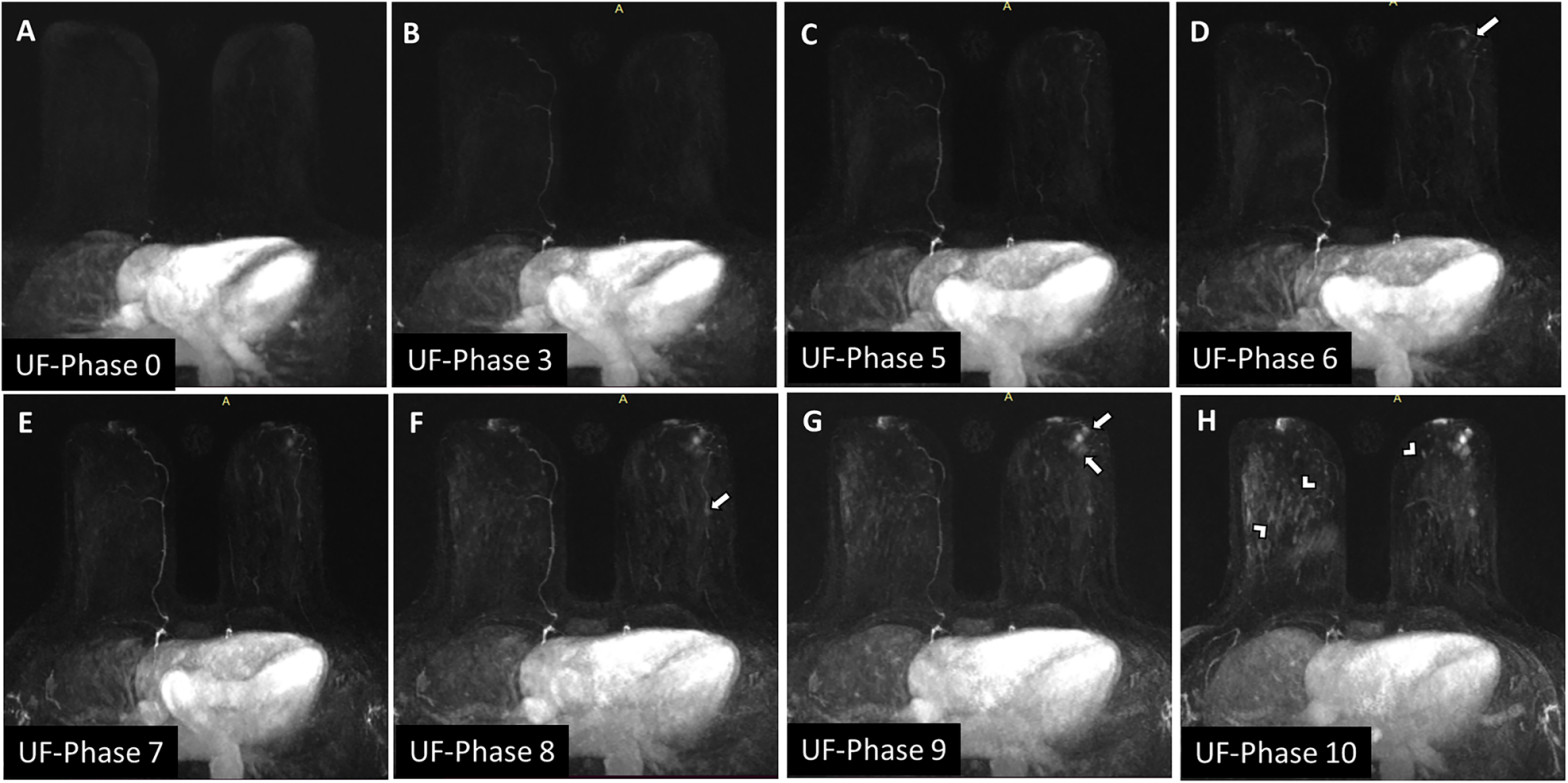
An example of how the IE-phase was calculated. **A**–**H** Sequentially subtracted ultrafast MIP images. **A** The phase at which the descending aorta begins to enhance was considered phase 0. **D** A left retroareolar circumscribed, rounded mass (arrow) was first enhanced at phase 6, so the IE-phase for that mass was 6. **F** A similar mass first enhanced as phase 8, so the IE phase for it was 8. **G** Two similar left retro areolar masses first enhanced at phase 9, so the IE-phase for them was 9. **H** Note the BPE (arrowheads) began to enhance at this late phase. The masses were pathologically proven to be papillomas

From the ultrafast curve, three parameters were manually calculated: TTE, MS, and the IE phase. We calculated TTE by visually determining the time interval between the start of descending thoracic aortic enhancement and the start of breast lesion enhancement on subtracted MIP images [4]. MS was calculated from the curve alone by determining the steepest part of the curve, and then the difference between the RE percentage at the two points of the steepest part was divided by the time interval [8] (Fig. 2). Regarding the IE phase, we considered the time point at which the descending aorta started to enhance on subtracted MIP images as phase zero, followed by sequential phases. The phase at which the lesion began to enhance visually was considered the IE phase [11] (Fig. 3).

**Fig. 4:**
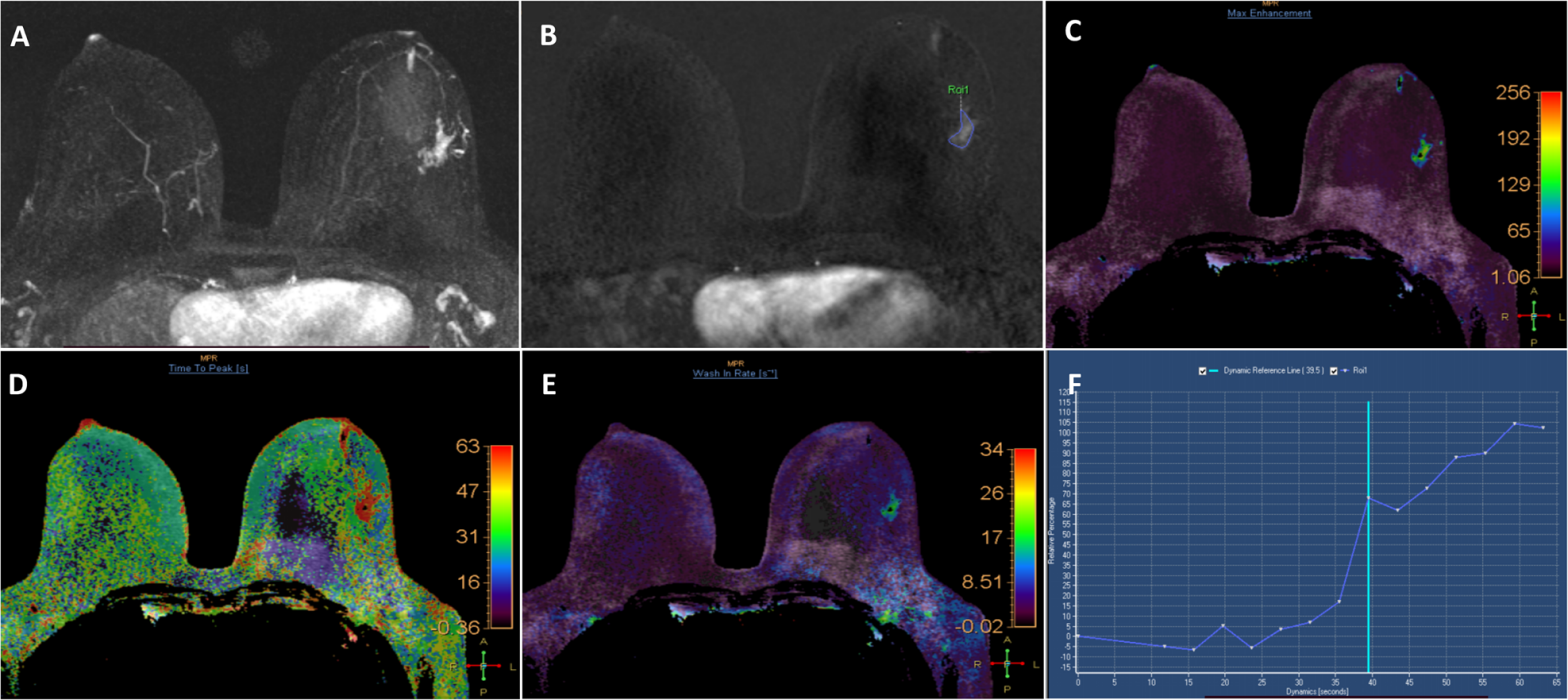
A 32-year-old female presented with a left breast mass (**A**), subtracted MIP image derived from ultrafast MRI at phase 13, showing an irregular mass with spiculated margin in the left lower outer quadrant. **B** Subtracted ultrafast image at the same phase showing freehand ROI outlining the lesion. **C**–**E** Semiautomatically generated color-coded maps for ME, TTP, and wash-in rate showing the previously described mass displaying different colors from the rest of the normal breast tissue. **F** The wash-in curve was generated once the ROI was placed on the lesion

Once the ROI was drawn manually, other parameters and their color maps (Fig. 4) were semiautomatically generated by our workstation, including RE, ME, MRE, TTP, and wash-in rate.

RE represents the percentage of enhancement compared to the non-contrast study. In our study, we evaluated the RE at phase 12. ME represents the peak enhancement of the lesion during the ultrafast sequences [11]. MRE was analyzed automatically using equation [13]. TTP represents the interval between the first ultrafast sequence and the lesion’s ME. The wash-in rate was calculated as the maximum gradient of the SI changed from the “time of arrival” to “time to peak” [14].

The manually generated parameters were combined (combination 1), and the semiautomatically generated parameters were combined (combination 2). The two radiologists correlated the ultrafast parameters results with the pathological results, and by ROC curve analysis, a cut-off value for each parameter was determined.

In a second interpretation session, a third radiologist, with 13 years of experience in breast imaging and blinded to the histopathological data, interpreted the ultrafast sequences. The radiologist was asked to characterize the lesions as benign or malignant depending on their morphological criteria. If the lesion’s morphology was indeterminate, he was asked to use the pre-determined cut-off values of the manually and semiautomatically generated parameters to categorize the lesion as either benign or malignant. If MS, RE, ME, MRE, and wash-in rate values of a lesion were higher than their cut-off values (7.25%/s, 51.4%, 71.5, 83.6% and 6.9/s, respectively) and TTE, IE phase and TTP value were equal to or lower than their cut off value (15.5 s, 4 s and 61 s, respectively), the lesion was categorized as malignant. When one or more of the parameters did not meet these criteria, the reader relied solely on MS and wash-in rate.

In a third interpretation session and after randomization of the study case registry, a fourth different radiologist with 15 years’ experience in breast imaging interpreted the dynamic contrast enhanced (DCE) MRI (without the ultrafast sequences), while still blinded to the histopathological results, and was asked to characterize the breast lesion into benign or malignant depending on morphologic criteria and the dynamic curve according to the last ACR-BIRADS lexicon [15].

### Statistical analysis

Data analysis was performed using SPSS software, version 25 (SPSS Inc., PASW Statistics for Windows version 25). Quantitative data were described as the mean ± standard deviation for normally distributed data and as the median (with minimum and maximum values) for non-normally distributed data. The significance of the obtained results was judged at the (≤ 0.05) level. The chi-square or Fisher’s exact test was used to compare qualitative data. The Mann–Whitney *U*-test was used to compare the two studied groups for non-normally distributed data. Student’s *t*-test was used to compare two independent groups for normally distributed data. The receiver operating characteristic curve (ROC curve) was used to determine the validity of continuous variables (sensitivity and specificity) by identifying the optimal cut-off point. Predictive values and accuracy were assessed using cross-tabulation. Univariate and multivariate regression analysis were used to assess the presence of dependent and independent risk predictors of malignancy. Interobserver agreement was assessed using intraclass correlation coefficients (ICCs) at 95% confidence intervals (CIs).

## Results

### Participant characteristics

This study included 264 patients; 189 (71.5%) were premenopausal and 75 (28.4%) were postmenopausal. The mean age of patients with benign lesions was 42.8 ± 11.9 years, and with malignant lesions was 45.9 ± 11.4 years (*p*-value = 0.051). The patients’ demographic data are presented in Table 2. This study included 273 breast lesions, as seven patients had multiple breast lesions (*N* = 16), while the remaining 257 patients had a solitary lesion. The breast lesions were divided into two groups according to their histopathological results: Benign (120/273 (44%)) and malignant (153/273 (56%)) lesions. Detailed pathological results are demonstrated in Table 2.

**Table 2:** Demographic criteria of the 264 participants and the final pathological result of the 273 lesions included in this study

Variables	Numbers (%)
All subjects (*n* = 264)	
Number of 1st-degree relatives with breast cancer	
0	194 (73.5%)
1	52 (19.7%)
2	10 (3.7%)
3	8 (3%)
Number of 2nd-degree relatives with breast cancer	
0	230 (87.1%)
1	26 (2.7%)
2	3 (1.1%)
3	5 (1.9%)
Hormonal status	
Pre-menopausal	189 (71.5%)
post-menopausal	75 (28.4%)
Lesions pathology	
Benign (*n* = 120)	
Fibrocystic disease	11 (4.1%)
Focal fibro-adenosis	12 (4.3%)
Fibroadenoma	21 (7.7%)
Papilloma	14 (5%)
Hamartoma	7 (2.6%)
Sclerosing adenosis	3 (1.1%)
Periductal mastitis	21 (7.7%)
Infected duct-ectasia	4 (1.5%)
Granulomatous mastitis	27 (10%)
Malignant (*n* = 153)	
Low-grade DCIS	8 (2.9%)
High-grade DCIS	7 (2.6%)
IDC	129 (47.2%)
ILC	9 (3.3%)

*DCIS* ductal carcinoma in situ, *IDC* invasive ductal carcinoma

### Lesions’ morphological MRI criteria

186 of the 273 breast lesions were described as masses, and 87 were described as non-mass enhancement (NME) according to their morphology in the subtracted ultrafast and MIP images. MRI criteria for the 264 patients, as well as the differences in MRI morphological criteria between benign and malignant lesions, are demonstrated in Supplementary Tables S1 and S2.

### Ultrafast kinetic parameters

#### Combination 1 (manually generated)

The mean interpretation time of the manually generated parameters, including ROI placement and parameter calculation, was 5 ± 2.7 min.

TTE and IE phases were lower in malignant lesions than in benign ones (*p*-value < 0.001). However, MS was higher in malignant lesions than in benign ones (*p*-value < 0.001 (Table 3).

**Table 3:** Comparison of different ultrafast kinetic parameters between benign and malignant lesions

Ultrafast parameter	Pathology		*p*-value
	Benign *N* = 120 (%)	Malignant *N* = 153 (%)	
Manually generated parameters			
▪ TTE (s)	20 (8–40)	8 (0–20)	< 0.001^*^
▪ IE phase	5 (2–8)	3 (0–16)	< 0.001^*^χ
▪ MS (%/s)	5 (1–17)	15 (2–140)	< 0.001^*^
Semi-automatically generated parameters			
▪ RE (%)	36 (3–180)	87 (2–635)	< 0.001^*^
▪ ME	63.6 (12–204)	103 (30–330)	< 0.001^*^
▪ MRE (%)	54 (3.5–222)	165 (31–640)	< 0.001^*^
▪ TTP (s)	63 (42–68)	59 (42–230)	0.002^*^
▪ Wash-in rate (/s)	5.9 (1.2–18)	9 (3–59)	< 0.001^*^

NB**:** parameters described as median (min-max). The test of significance is the Mann–Whitney test, except *χ* represents the Chi-Square test

*TTE* time to enhancement, *IE* initial enhancement, *MS* maximum slope, *RE* relative enhancement, *ME* maximum enhancement, *MRE* maximum relative enhancement, *TTP* time to peak

^*^ Statistically significant

**Fig. 5:**
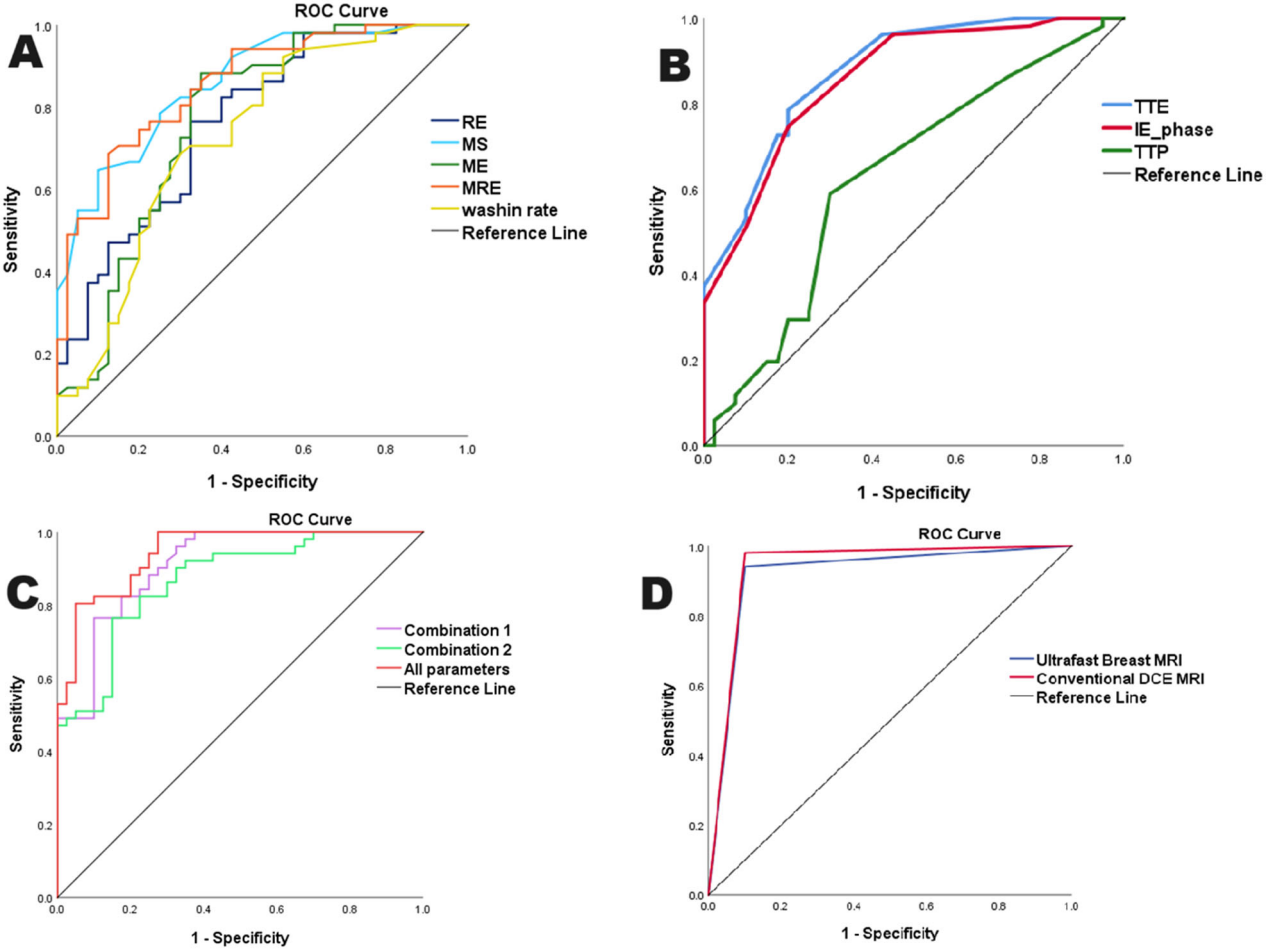
Roc curve analysis: **A** of MS, RE, ME, MRE, and wash-in curve; **B** of TTE, IE phase, and TTP; **C** of combination 1, 2, and all parameters combined; **D** of ultrafast MRI and conventional DCE-MRI

Among manual ultrafast parameters analyzed, the MS showed the highest sensitivity (82.4%); however, TTE and IE phases showed the same highest specificity (80%). On ROC curve analysis, the TTE, IE phase, and MS cut-off values of (≤ 15.5 s, ≤ phase 4, and > 7.25%/s) showed area under the curve (AUC) of (0.876, 0.858, and 0.858), respectively (Table 4 and Fig. 5).

**Table 4:** Validity of the ultrafast kinetic parameters in differentiating between benign & malignant lesions

	AUC	95% CI	*p*-value	Cut-off point	Sensitivity %	Specificity %	PPV%	NPV%	Accuracy%
Manually generated parameters:									
▪ TTE (s)	0.876	0.841–0.910	< 0.001^*^	15.5	78.4	80.0	83.3	74.4	79.1
▪ MS (%/s)	0.860	0.824–0.897	< 0.001^*^	7.25	82.4	70.0	77.8	75.7	76.9
▪ IE phase	0.858	0.820–0.895	< 0.001^*^	4	74.5	80.0	83	70	78.0
▪ Combination 1	0.910	0.876–0.937	< 0.001^*^	–	76.5	90	90.7	79.2	82.2
Semi-automatically generated parameters:									
▪ RE (%)	0.766	0.718–0.815	< 0.001^*^	51.4	82.4	60.0	72.4	72.7	72.5
▪ ME	0.769	0.717–0.820	< 0.001^*^	71.5	88.20	65.0	76.3	81.2	78.0
▪ MRE (%)	0.855	0.818–0.893	< 0.001^*^	83.6	84.3	67.5	73.7	56.6	64.4
▪ TTP (s)	0.630	0.571–0.688	< 0.001^*^	61.0	58.8	70.0	71.4	57.1	63.7
▪ Wash in rate (/s)	0.729	0.675–0.782	< 0.001^*^	6.9	80.4	52.5	68.3	67.7	68.1
▪ Combination 2	0.869	0.830–0.902	< 0.001^*^	–	76.5	85	86.7	73.9	79.8
▪ All parameters combined (combination1 + 2)	0.944	0.915–0.965	< 0.001^*^	–	80.4	95	95	79.2	86.8

NB: combination 1 = TTE + MS + IE phase, Combination 2 = RE + ME + MRE + TTP + wash-in rate

*TTE* time to enhancement, *IE* initial enhancement, *MS* maximum slope, *RE* relative enhancement, *ME* maximum enhancement, *MRE* maximum relative enhancement, *TTP* time to peak

^*^ Statistically significant

There was no significant difference regarding TTE, MS, or IE phase between invasive cancers and ductal carcinoma in situ (DCIS) (*p*-value 0.109, 0.504, 0.202, respectively).

#### Combination 2 (semiautomatically generated)

The mean interpretation time for the semiautomatically generated parameters, including ROI placement and parameter generation, was 1 ± 0.5 min.

RE, ME, MRE, and wash-in rates were significantly higher in malignant lesions than in benign ones (*p*-value < 0.001 for all). However, TTP was lower in malignant lesions than in benign ones (*p*-value 0.002) (Table 3).

Among semiautomatically derived ultrafast parameters, ME showed the highest sensitivity (88.2%), while TTP showed the highest specificity (70%). RE, MRE, and wash-in rate cut-off values > (51.4, 83.6%, and 6.9 s) showed a sensitivity of (82.4%, 84.3%, and 80.4%) and specificity of (60%, 67.5%, and 52.5%), respectively, in discriminating benign from malignant lesions (Table 4 and Fig. 5).

On comparing the semiautomatically generated parameters between invasive cancers and DCIS, we found no significant difference regarding RE, ME, MRE, and wash-in rates (*p*-value 0.395, 0.362, 0.798, and 0.256, respectively).

#### The performance of the combined ultrafast parameters (manually and semiautomatically generated)

On ROC curve analysis, the combination of the manually vs semiautomatically generated parameters (combination 1 vs combination 2) showed (AUC = 0.910 vs 0.869, sensitivity = 76.5% vs 76.5%, and specificity = 90% vs 85%) in discriminating between benign from malignant lesions, respectively. On combining all parameters together (combination 1 + combination 2), the AUC rose to 0.944, sensitivity rose to 80.4%, and specificity rose to 85% in discriminating the two groups (Table 4 and Fig. 5C). The AUC differences between the two groups are descriptive only and were not statistically tested.

#### Regression analysis

The logistic regression analysis model was run to ascertain the effects of the ultrafast parameters on the likelihood of malignancy. In Table 5, all eight predictor variables (MS, TTE, IE phase, RE, ME, MRE, TTP, and wash-in rate) were statistically significant on the univariable analysis. So, they were all entered in a multivariable logistic regression.

**Table 5:** Univariate and multivariate regression analysis for prediction of malignancy

Predictors	Univariate regression				Multivariate regression			
	*p*-value	Odds ratio	95% CI for odds ratio		*p*-value	Odds ratio	95% CI for odds ratio	
			Lower	Upper			Lower	Upper
Manually generated parameters:								
TTE	< 0.001^*^	0.759	0.716	0.805	0.248	0.780	0.512	1.188
MS	< 0.001^*^	1.304	1.233	1.379	< 0.001^*^	1.258	1.108	1.428
IE phase	< 0.001^*^	0.351	0.281	0.438	0.001^*^	0.538	0.110	0.702
Semi-automatically generated parameters:								
RE	< 0.001^*^	1.017	1.012	1.021	0.068	1.016	0.995	1.032
ME	< 0.001^*^	1.019	1.014	1.024	0.046^*^	1.048	1.029	1.059
MRE	< 0.001^*^	1.023	1.018	1.027	0.042^*^	1.040	1.019	1.060
TTP	0.002^*^	0.942	0.908	0.977	0.594	1.021	0.947	1.100
Wash in rate	< 0.001^*^	1.182	1.116	3.172	< 0.001^*^	1.687	1.123	1.967

*CI* confidence interval, *TTE* time to enhancement, *IE* initial enhancement, *RE* relative enhancement, *ME* maximum enhancement, *MRE* maximum relative enhancement, *TTP* time to peak

^*^ Statistically significant

Of the eight predictor variables, only MS, IE phase, ME, MRE, and wash-in rate maintained their significance in the multivariate analysis as predictors of malignancy. Breast lesion with an IE phase higher than 5 has a 0.538 times lower odds ratio of being malignant. However, breast lesion with MS of more than 7.25%/s, ME of more than 71.5, MRE of more than 83.5% and wash-in rate of more than 6.9/s has 1.258-, 1.048-, 1.040-, and 1.687-times higher odds to be malignant, respectively. MS and wash-in rate showed the highest significance and the highest probabilities to predict malignancies (*p*-value < 0.001).

#### Comparison between ultrafast breast MRI and conventional DCE-MRI performance

**Fig. 6:**
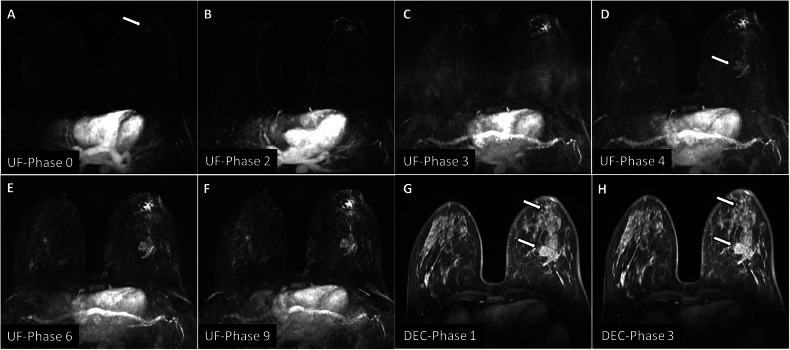
An example of how ultrafast MRI can increase lesion visibility compared to the conventional DCE-MRI. **A**–**F** Sequentially subtracted ultrafast MIP images. **A** A left retro-areolar irregular spiculated mass first appeared at phase 0 (arrow). **D** A second irregular mass first appeared at phase 4 (arrow), seen at the lower outer quadrant of the left breast. **G**, **H** The corresponding conventional DCE-MRI at phases 1 and 3, respectively, shows that the breast is dense with marked BPE obscuring the two masses (arrows)

Using combined ultrafast parameters (manually and semiautomatically generated), the reader correctly diagnosed 94.1% (144/153) of malignant lesions and 90% (108/120) of benign lesions, while missing 5.9% (9/153) of malignant lesions (5 low-grade DCIS, 1 grade 1 IDC, and 2 invasive lobular carcinoma (ILC) lesions) and falsely misdiagnosed 10% (12/120) benign lesions as malignant ones (6 papilloma, 4 granulomatous mastitis and 2 sclerosing adenosis). By interpreting the DCE-MRI, the reader correctly diagnosed 98% (150/ 153) of malignant lesions and 90% (108/120) of benign lesions, missing 2% (3/153) of malignant lesions and falsely misdiagnosing 10% (12/120) of benign lesions as malignant. Ultrafast and DCE-MRI showed the same specificity (90%); however, DCE-MRI showed higher sensitivity (98%) than ultrafast MRI (94.1%). Both techniques effectively detected malignancy with an AUC of 0.921 and 0.940 for ultrafast and DCE-MRI, respectively (Fig. 5 and Supplementary Table S3). Figure 6 illustrates how BPE can obscure lesions and how ultrafast MRI can enhance lesion visibility.

#### Interobserver reliability of the ultrafast kinetic parameters

The interobserver agreement of the ultrafast kinetic parameters between the two radiologists who performed the first reading session is summarized in the Supplementary Table (S4). We found strong agreement between the two readers regarding all ultrafast parameters except for the MS parameter, which showed moderate agreement between the two readers (ICC = 0.758 and 95% CI = 0.484–0.897).

## Discussion

The main finding of this study is that adding semiautomatically generated parameters to the more popular parameters, TTE and MS, increases the overall discriminating power of ultrafast MRI in characterizing breast lesions. To the best of our knowledge, this is the first study to evaluate the efficacy of semiautomatically generated parameters derived from ultrafast MRI in discriminating between benign and malignant lesions, as well as the effect of adding these parameters to the popular TTE and MS parameters on the overall efficacy of the ultrafast protocol. These parameters were easily derived, rapidly generated, and less complex. They can also be generated routinely by workstations without the need for special software that may only be available in a few MRI centers.

Several studies have evaluated various abbreviated MRI protocols, including ultrafast sequences, aiming to reduce both scan and interpretation times without compromising sensitivity and specificity [16–19]. In this study, the ultrafast TWIST sequence scan time was only 77 s, giving us morphologic and kinetic data from subtracted and MIP images. The interpretation time of the semiautomatically generated parameters was significantly shorter than that of the manually generated ones (1 ± 0.5 vs 5 ± 2.7 min, respectively). Although the AUC difference between manual and semiautomatic parameters is very small (AUC = 0.910 vs 0.869), this time-saving measure can enhance patient accessibility to breast MRI, particularly in high-volume clinical settings where resource constraints are common. Furthermore, the reduction in the acquisition and interpretation times can improve cost-effectiveness by optimizing resource utilization and increasing throughput. Further studies are needed to gain a better understanding of the clinical and economic implications of using ultrafast MRI. An additional advantage of the semiautomatically generated parameters is their operator independence, which helps reduce the likelihood of human error, enhances consistency and reproducibility, unlike manually generated parameters that are subject to individual errors depending on the expertise of the operator, making it more biased and less reliable.

In agreement with the previous studies [17, 20], non-circumscribed margins of masses, including irregular and speculated margins, and segmental clumped NME were the most significant morphologic criteria in malignant lesions included in this study. In our study, if the lesion’s morphology was indeterminate, we used the manually and semiautomatically generated ultrafast parameters cut-off values to categorize the lesion as either benign or malignant. However, when one or more of the parameters did not meet the cutoff values, we relied solely on MS and wash-in rate. We relied on this ‘rule of thumb’ as a practical approach to aid clinical decision-making in ambiguous and indeterminate lesions. Further expanding on this, the ‘rule of thumb’ can minimize false-negative or positive results in challenging cases, thereby increasing overall diagnostic performance and potentially improving clinical confidence and patient management.

The results of TTE have been controversial in the literature, as some studies have reported that TTE can accurately discriminate between benign and malignant lesions [3, 4, 21]. However, others found no statistically significant difference between the two groups [22]. This controversy can be attributed to the differences in temporal resolution and time intervals between sequential ultrafast sequences. Additionally, the different methods of TTE calculation are either visual, manual, or semiautomated. In our study, the TTE cut-off value of ≤ 15.5 s showed the best performance among manually generated parameters in discriminating between benign and malignant lesions (AUC = 0.876). On the other hand, an MS cut-off value of > 7.25% showed excellent performance in discriminating between benign and malignant lesions (AUC = 0.86). This cut-off value is very close to that reported by Goto et al (7.3%/s) and slightly higher than that reported by Mann et al (6.4%/s) even though the two studies used 3 T MRI machines [20, 23]. Malignant lesions avidly enhance compared to benign ones, secondary to the angiogenesis that occurs in malignant lesions [16, 24]. Milon et al reported significantly higher ME in malignant lesions than in benign ones [4]. In concordance with these studies, we found that RE, ME, wash-in rate, and MRE were significantly higher in malignant lesions compared to benign ones, with the MRE cut-off value > 83.6% having the best performance among them (AUC = 0.855). In multivariate regression analysis, MS and wash-in rate showed the highest significance among all parameters (*p*-value < 0.001). However, the wash-in rate showed a wider 95% CI compared to MS (1.687–1.123 vs 1.258–1.108, respectively). This can be explained by the study population and lesion nature variability, as our study included variable benign and malignant pathologies, which may vary in the degree of blood uptake in the early phase. This interesting finding warrants further exploration of the wash-in rate values among different grades and molecular subtypes of breast cancer in future studies.

Previous studies evaluated the role of ultrafast parameters in upgrading DCIS, and they reported that TTE, MS, and ME can differentiate between invasive and in situ carcinomas [25, 26]. Unlike the previously mentioned studies, we found that ultrafast parameters did not show a statistically significant difference between invasive cancers and DCIS. This can be attributed to the small percentage of DCIS lesions included in this study (5.5% of all lesions).

In our study, adding semiautomatic parameters (combination 2) improved the discriminating power of the ultrafast MRI compared to combination 1 alone, with sensitivity, specificity, and AUC (80.4% vs 76.5%, 95% vs 90%, and 0.994 vs 0.91, respectively). To our knowledge, the discriminating power of the combined parameters in our results is novel and has not been previously reported in the literature. In concordance with our results, a recent meta-analysis analyzed 16 studies aiming to assess the efficacy of ultrafast MRI in discriminating between benign from malignant breast lesions. Those 16 studies mainly used the two commonly used ultrafast kinetic parameters, MS and TTE. The previous meta-analysis concluded that ultrafast breast MRI has a pooled sensitivity of 83%, a pooled specificity of 77% and a pooled AUC of 0.876 in differentiating the two groups [27].

Although the overall sensitivity of different abbreviated MRI protocols is very close to that of the conventional full MRI protocol, a small but measurable loss in sensitivity is observed across multiple studies. This was explained by the presence of a small number of false negative results caused by low-grade DCIS or certain types of lobular carcinomas, which tend to enhance later in delayed images [24]. In line with further studies [8, 24], both ultrafast MRI and conventional DCE-MRI used in our study demonstrated excellent performance in characterizing breast lesions. However, the ultrafast protocol showed lower sensitivity as it missed nine breast cancer lesions out of the 153 malignant lesions, which were in situ carcinomas or lower-grade malignancies (grade 1 IDC or ILC). From these nine lesions, DCE-MRI missed only three malignant lesions and misdiagnosed them as benign ones.

This study has several limitations, as it is a single-center study with a relatively small group of patients. A relatively small number of DCIS cases were included in this study, making the evaluated efficacy of the ultrafast parameters in upgrading DCIS into invasive cancers inaccurate. Since this study focuses on the discrimination between benign and malignant breast lesions, we did not compare the efficacy of the ultrafast parameters between mass and NME lesions. We also did not evaluate the efficacy of the ultrafast parameters in discrimination between several molecular subtypes of breast cancers.

In conclusion, ultrafast MRI appears to be the future of breast MRI, as it can provide accurate morphologic and kinetic data, similar to those obtained with the conventional DCE-MRI protocol. Depending on our results, we recommend adding semiautomatic kinetic parameters to the commonly used MS and TTE derived from ultrafast breast MRI to achieve the highest discriminating power between benign and malignant lesions.

## Supplementary information

ELECTRONIC SUPPLEMENTARY MATERIAL

## Data Availability

All data generated or analyzed during this study are included in this published article.

## Abbreviations

AUC: Area under the curve
BPE: Background parenchymal enhancement
CI: Confidence interval
DCE: Dynamic contrast enhanced
DCE-MRI: Dynamic contrast-enhanced MRI
DCIS: Ductal carcinoma in situ
IDC: Invasive ductal carcinoma
IE: Initial enhancement
ILC: Invasive lobular carcinoma
ME: Maximum enhancement
MIP: Maximum intensity projection
MRE: Maximum relative enhancement
MRI: Magnetic resonance imaging
MS: Maximum slope
NME: Non-mass enhancement
RE: Relative enhancement
ROC: Receiver operating curve
ROI: Region of interest
TTE: Time to enhancement
TTP: Time to peak
TWIST: Time-resolved angiography with stochastic trajectories
VIBE: Volume interpolated breath-hold examination
