# Supplementary material for: Diagnostic performance of kinetic parameters derived from ultrafast breast MRI in characterizing benign and malignant breast lesions: the added value of the semiautomatically based parameters
Source: Insights Imaging. 2025 Dec 22;16:286. doi: 10.1186/s13244-025-02162-8 (PMC12722194; doi:10.1186/s13244-025-02162-8)
Supplement: Supplementary file 1 — ELECTRONIC SUPPLEMENTARY MATERIAL [file 13244_2025_2162_MOESM1_ESM.pdf]

Diagnostic performance of kinetic parameters derived from ultrafast breast MRI in characterizing benign and malignant breast lesions: The added value of the semiautomatically based parameters

ELECTRONIC SUPPLEMENTARY MATERIAL

Table (S1): MRI criteria of the 264 patients included in this study:

**Variables**	**Numbers (%)**
All subjects (n=264)	
**Amount of FGT**	
▪ Fatty	26 (9.8%)
▪ Scattered FG	91 (34.5%)
▪ Heterogenous FG	94 (35.6%)
▪ Extreme FG	53 (20.1%)
**BPE symmetry**	
• Symmetrical	170 (64.4%)
• Asymmetrical	94 (35.6%)
**BPE level**	
• Minimal	29 (11%)
• Mild	111 (42%)
• Moderate	109 (41.3%)
• Marked	15 (5.7%)
**Presence of enhancing foci:**	
• Present	97 (36.7%)
• Absent	167 (63.3%)

NB: FGT: fibro glandular tissue, BPE: background parenchymal enhancement.

Table (S2): Comparison between MRI morphologic features of benign vs. malignant lesions:

Characteristic	Benign (n=120)	Malignant (n=153)	P-value
**Mass Lesions (n=186)**	**(N=69) (%)**	**(N=117) (%)**	
▪ **Shape**			0.792
**Irregular**	36 (52.2%)	66 (56.4%)	
**Rounded**	18 (26.1%)	30 (25.6%)	
**Oval**	15 (21.7%)	21 (17.9%)	
▪ **Margin**			<0.001*
**Circumscribed**	36 (52.1%)	14 (11.9%)	
**Speculated**	3 (4.3%)	47 (40.1%)	
**Irregular**	30 (43.5%)	56 (47.8%)	
▪ **Enhancement pattern**			0.036*
**Homogeneous**	18 (26.1%)	40 (31.2%)	
**Heterogeneous**	12 (17.4%)	48 (25.8%)	
**Rim**	24 (34.8%)	49 (26.3%)	
**Dark internal septations**	15 (21.7%)	31 (16.7%)	
**NME lesions (n=87)**	**(N=51) (%)**	**(N=36) (%)**	
▪ **Distribution**			
**Focal**	7 (13.7%)	5 (13.9%)	
**Linear**	8 (15.7%)	4 (11.1%)	0.009*
**Segmental**	7 (13.7%)	16 (44.4%)	
**Regional**	23 (45.1%)	11 (30.6%)	
**Multiple regions**	6 (11.8%)	0 (0%)	
▪ **Enhancement pattern**			0.011*
**Homogeneous**	16 (31.4%)	15 (41.7%)	
**Heterogeneous**	22 (43.1%)	5 (13.9%)	
**Clumped**	13 (25.5%)	16 (44.4%)	

Notes: Data are N (%). The test of significance is Fisher's exact test. *statistically significant. NME: non-mass enhancement.

Table (S3): Comparison between diagnostic performance of Ultrafast MRI and conventional DCE-MRI in detection of breast cancer:

Measure	Ultrafast MRI	Conventional DCE-MRI
**Sensitivity**	94.1%	98%
**Specificity**	90%	90%
**PPV**	92.3%	92.5%
**NPV**	92.3%	97.3%
**AUC**	0.921	0.940
**Accuracy**	92.3%	94.5%
**false positive**	12	12
**False negative**	9	3
**True positive**	144	150
**True negative**	108	108

NB: DCE-MRI= Dynamic Contrast Enhanced MRI

Table (S4): Interobserver reliability of the ultrafast kinetic parameters:

Ultrafast parameter:	Agreement degree	ICC	95% CI
**TTE**	Strong	0.911	0.848-0.949
**MS**	Moderate	0.758	0.484-0.897
**IE phase**	Strong	0.953	0.918-0.974
**RE**	Strong	0.862	0.652-0.946
**ME**	Strong	0.922	0.866-0.955
**MRE**	Strong	0.959	0.928-0.977
**TTP**	Strong	0.805	0.656-0.889
**wash in rate**	Strong	0.865	0.773-0.921

NB: ICC: interclass agreement coefficient, CI: confidence interval, TTE: time to enhancement, IE: initial enhancement, MS: maximum slope, RE: relative enhancement, ME: maximum enhancement, MRE: maximum relative enhancement and TTP: time to peak.
